# Baseline immunoglobulin G and immune function in non-Hodgkin lymphoma: a retrospective analysis

**DOI:** 10.3389/fimmu.2024.1334899

**Published:** 2024-04-30

**Authors:** Danielle Brazel, Christopher Grant, Angelo Cabal, Wen-Pin Chen, Lauren Pinter-Brown

**Affiliations:** ^1^ Department of Hematology/Oncology, Scripps Clinic/Scripps Green Hospital, La Jolla, CA, United States; ^2^ Department of Medicine, University of California Irvine Medical Center, Orange, CA, United States; ^3^ Department of Biostatistics, University of California Irvine Medical School, Irvine, CA, United States; ^4^ Chao Family Comprehensive Cancer Center, University of California Irvine, Orange, CA, United States

**Keywords:** non-Hodgin lymphoma, immune function, IgG, rituximab, intravenous immunoglobulin (IVIG), indolent lymphoma, aggressive lymphoma, hospitalization - statistics and numerical data

## Abstract

**Introduction:**

Non-Hodgkin’s lymphoma (NHL) encompasses a diverse group of lymphoma subtypes with a wide range in disease course. Previous studies show that hypogammaglobulinemia in treatment-naïve patients is associated with poorer survival in high grade B-cell non-Hodgkin’s lymphomas, though it is not known how this applies across all B-cell lymphoid malignancies.

**Methods:**

We conducted a retrospective study of immunoglobulin levels and clinical outcomes including survival, hospitalization, and infection rates in patients diagnosed with B-cell non-Hodgkin lymphomas of all grades at our institution.

**Results:**

Two-hundred twenty-three adults (aged = 18 years) with available pre-treatment IgG levels were selected, with hypogammaglobulinemia defined as IgG< 500 mg/mL. For this analysis, we grouped DLBCL (n=90), Primary CNS (n=5), and Burkitt lymphoma (n=1) together as high-grade, while CLL (n=52), mantle cell (n=20), marginal zone (n=25), follicular (n=21), and Waldenstrom macroglobulinemia (n=5) were low-grade. The incidence of hypogammaglobulinemia in our cohort of both high and low-grade lymphoma patients was 13.5% (n=30). Across all NHL subtypes, individuals with baseline IgG< 500 mg/dL showed an increased rate of hospitalization (4.453, CI: 1.955-10.54, p= 0.0005) and higher mortality (3.325, CI: 1.258, 8.491, p= 0.013), yet no association in number of infections when compared with those with IgG=500 mg/dL. There was a higher hospitalization rate (3.237, CI: 1.77-6.051, p=0.0017) in those with high-grade lymphoma with hypogammaglobulinemia when compared with low-grade. There was no statistically significant difference in individuals who were alive after three years in those with baseline IgG<500 mg/dL.

**Discussion:**

Our study is the first to analyze incidence of hypogammaglobulinemia at the time of diagnosis of NHL as a potential biomarker of interest for future outcomes including hospitalization and infection.

## Introduction

Several retrospective analyses explored biomarkers to estimate host immune homeostasis (such as lymphocyte count or immunoglobulin (Ig) levels) and tumor microenvironment (i.e. monocyte count or tumor-associated macrophages). Absolute lymphocyte count at diagnosis and recovery of absolute lymphocyte count after anticancer treatment have been used as surrogate markers of host immune status (1). In follicular lymphoma (2) and diffuse large B cell lymphoma (DLBCL) (1, 3) absolute lymphocyte-monocyte ratio is a prognostic factor for progression-free survival (PFS) and overall survival (OS). One study found low lymphocyte-monocyte ratio associated with worse 1-year PFS (4).

Previous studies found non-Hodgkin lymphoma (NHL) associated with hypogammaglobulinemia in treatment-naïve patients (5) is an adverse prognostic biomarker (6, 7). One study found the incidence of hypogammaglobulinemia (defined as IgG <700 mg/dL, IgM <40 mg/dL, IgE <2Ul/mL, and IgA <70 mg/dL) is 22.1% in newly diagnosed, treatment naïve patients with DLBCL (8). The most common deficiency is IgG (13.5%) though any deficiency was associated with worse event-free survival (HR 1.94, 95% CI 1.16-3.24) and worse overall survival (OS) (HR 2.02, 95% CI 1.17-3.49), independent of international prognostic index (IPI) (8).

Existing literature demonstrates an association between hypogammaglobulinemia in treatment-naïve patients with DLBCL and poor OS. The same relationship has not been explored in a larger group of lymphomas to compare and contrast differences across subtypes. The present study assesses rates of baseline hypogammaglobulinemia and clinical outcomes across the spectrum of B-cell NHLs on a number of clinical outcomes.

## Methods

We performed a single center retrospective analysis in B-cell NHL patients evaluated at our institution from January 2010 to June 2022. Eligibility criteria for our cohort were adults at least 18 years of age with histopathologically proven non-Hodgkin lymphomas including DLBCL, chronic lymphocytic leukemia (CLL), mantle cell lymphoma, marginal zone lymphoma, follicular lymphoma, primary CNS lymphoma, and Waldenstrom macroglobulinemia with a baseline IgG taken within 2 years of diagnosis and initiation of treatment. Pre-treatment IgG levels were recorded and patients were treated per standard of care regimen if clinically indicated for their disease type and status.

Baseline clinical, staging, and laboratory information was collected, including hospital admissions, date of death, and number of infections (specifically COVID-19). Hypogammaglobulinemia was defined as serum IgG level less than 500 mg/dL. Details pertaining to treatment with rituximab or intravenous immunoglobulin (IVIG) was recorded. Immune function was measured as number of hospitalizations for infections, number of outpatient healthcare encounters related to infections, and COVID-19 infection after NHL diagnosis were also recorded. Preliminary results for overall survival, 3-year survival, and 5-year survival were also analyzed.

Population baseline characteristics were analyzed via univariate logistic models for categorical variables. Results of multivariate logistic models were used to predict outcomes for baseline IgG dichotomized at 500 mg/dL in high versus low grade cancers as well as for dichotomized baseline IgG and treatment with either rituximab or IVIG. Analysis for covariates’ effects in 7 negative binomial models with key covariates were used for number of infections and number of hospitalizations within the patient population. OS was calculated with a joint hypothesis testing utilizing the F-statistic in 7 Cox models. For all analysis, Global Likelihood Ratio tests were utilized to examine whether the current model is a good fit to the data utilizing a p-value of 0.05.

Descriptive analysis via SAS version 9.4 was utilized to summarize the population baseline characteristics. Univariate and multivariate logistic regressions were conducted to study the associations between each of the binary outcomes and covariates which include the dichotomized baseline IgG at 500 mg/dL, high or low grade cancer, and type of treatments. Negative binomial regression models were utilized to evaluate the associations between a count outcome and covariates. The Kaplan-Meier estimates and Cox model were performed to analyze the overall survival data.

## Results

### Descriptive statistics

Of the 500 NHL patients newly diagnosed at our medical center between January 2010 and June 2021, 223 met inclusion criteria including DLBCL (n=90), chronic lymphocytic leukemia (CLL, n=52), mantle cell lymphoma (n=20), marginal zone lymphoma (n=25), follicular lymphoma (n=21), primary CNS lymphoma (n=5), and Waldenstrom macroglobulinemia (n=5). A portion of the initial sample was excluded for lack of documented pre-treatment IgG level (n=220). In the context of our evaluation, individuals with DLBCL, primary CNS lymphoma, and 1 patient with Burkitt lymphoma were defined as high grade cancer, while more indolent cancers, such as Waldenstrom macroglobulinemia, CLL, mantle zone lymphoma, and follicular lymphoma were defined as low grade cancer. Within the study population, 13.5% (30/223) had hypogammaglobulinemia at diagnosis prior to treatment initiation. The incidence was similar within patients with high vs low grade lymphoma (13.5% and 13.4% respectively). On further analysis of 30 patients with baseline hypogammaglobulinemia, 13 patients (43.3%) had high grade B-cell lymphoma while 17 (56.7%) had low grade B-cell lymphoma (**Table 1**). In our sample of patients with lgG>500 at diagnosis, 83 (43%) had high grade cancer while 110 (57%) had low grade cancer (**Table 2**). These findings were not statistically significant (p=0.9731; **Table 2**).

**Table 1:** Descriptive statistics in categorical variables among 223 participants.

Characteristic		
High or Low Grade	high	96/223 (43%)
	low	127/223 (57%)

**Table 2:** Comparing lymphoma grade and IgG level around time of diagnosis.

Malignancy Grade	Baseline IgG < 500	Baseline IgG ≥ 500	P-value
High	13 (43.3%)	83 (43%)	0.9731
Low	17 (56.7%)	110 (57%)	

Within our sample the median age was 64 years (range 19-94). Baseline labs analyzed prior to initiation of anti-cancer treatment include a median LDH of 207 (67-4524). Treatment regimens include IVIG (n=23/223, 10.3%) and rituximab (n=147, 65.9%).

### Hospitalization rates

Across all types of B-cell NHL, individuals with baseline IgG <500 mg/dL demonstrated a 4.453 (95% CI 1.955, 10.54) times higher rate of hospitalization (p=0.0005) when accounting for grade of lymphoma (**Table 3**). Patients with high grade B-cell lymphoma demonstrated a 3.237 (95% CI 1.77, 6.051) times higher rate of hospitalization when compared to patients with a low grade B-cell lymphoma (p=0.000172) (**Table 3**). These results continued to be statistically significant when accounting for treatment. When adjusting for treatment with IVIG and rituximab, the odds of being hospitalized in patients with an IgG <500 mg/dL baseline is 3.754 (95% CI 1.575, 9.26) times higher than that in the patients whose baseline IgG>500 mg/dL (p=0.003161) (**Table 4**). When adjusting for treatment with IVIG and rituximab high grade B-cell lymphoma patients were 2.76 (95% CI 1.43, 5.451) times more likely to be hospitalized than that in patients with low grade B-cell lymphoma (p=0.002843) (**Table 4**). There was no statistical difference in patients treated with IVIG only, rituximab only, or a combination (p=0.141792). Among patients with baseline IgG<500 mg/dL and high grade B-cell lymphoma, there was a 2.119 (95% CI 0.524, 8.57) times incident rate of hospitalization when comparing high grade B-cell lymphoma patients with IgG>500 mg/dL when adjusting for treatment (p=0.29) that was not statistically significant. After adjusting for treatment effect, among patients with baseline IgG< 500 mg/dL and low grade B-cell lymphoma there was a 2.844 (95% CI 0.769, 10.517) times incident rate of hospitalization when comparing low grade B-cell lymphomas with baseline IgG>500 mg/dL (p=0.1171).

**Table 3:** Rate of hospitalization in high grade lymphoma.

Outcome	Effect	P-value	Odds Ratio (95%CI)
Hospitalization	High versus low grade lymphoma	**0.000172**	**3.237 (1.77, 6.051)**
Hospitalization	IgG <500 mg/dL and high grade lymphoma	**0.000465**	**4.453 (1.955, 10.54)**
Deceased	High versus low grade lymphoma	**0.000364**	**4.584 (2.051, 11.13)**
Decreased	IgG <500 mg/dL and high grade lymphoma	**0.012663**	**3.325 (1.258, 8.491)**
Alive at 3 years	High versus low grade lymphoma	**0.000301**	**0.168 (0.06, 0.424)**
Alvie at 3 years	IgG <500 mg/dL and high grade lymphoma	0.684391	0.791 (0.26, 2.588)

The bold values indicate significant findings.

**Table 4:** Results of multivariate logistic models for 3 categorical variables to predict an outcome among 223 patients whose data is available.

Outcome	P-Value	Effect	Estimated Odds Ratio & 95% CO
COVID Infections	0.72666	IgG <500 vs IgG >500	0.81 (0.215, 2.403)
	0.611223	IgG <500 and high grade lymphoma	1.233 (0.549, 2.785)
	0.329092	IgG <500 and high grade lymphoma and IVIG vs no therapy	3.646 (0.43, 25.717)
		IgG <500 and high grade lymphoma and Rituximab vs no therapy	0.885 (0.378, 2.112)
		IgG <500 and high grade lymphoma and IVIG and Rituximab vs no therapy	0.287 (0.015, 1.729)
Hospitalization	**0.003161**	**IgG <500 vs IgG >500**	**3.754 (1.575, 9.26)**
	**0.002843**	**IgG <500 and high grade lymphoma**	**2.76 (1.43, 5.451)**
	0.141792	IgG <500 and high grade lymphoma and IVIG vs no therapy	0.807 (0.037, 6.82)
		IgG <500 and high grade lymphoma and Rituximab vs no therapy	1.508 (0.702, 3.343)
		IgG <500 and high grade lymphoma and IVIG and Rituximab vs no therapy	4.296 (1.24,1 5.752)

The bold values indicate significant findings.

### Infections

Our results found no association between the number of healthcare encounters for infections and COVID-19 infections and baseline IgG level dichotomized at 500 (p=0.09). Among patients with baseline IgG<500 mg/dL, the incident rate of infections for the patients with high grade B-cell lymphoma is 10.123 (95% CI 3.274, 31.301) times the incident rate for the patients with low grade B-cell lymphoma (p=<0.0001) when adjusted for treatment with IVIG or rituximab. Among patients with baseline IgG>500 mg/dL, the incident rate of infections for patients with high grade B-cell lymphoma was 2.663 (95% CI 1.567, 4.526) times higher than patients with low grade B-cell lymphoma when adjusted for treatment with IVIG or rituximab (p=0.0003). Among those with high grade B-cell lymphoma, the incident rates of infections for those with baseline IgG<500 mg/dL is 1.943 (95% CI 0.805, 4.688) times the incident rate for the those with a baseline IgG >500 mg/dL (p=0.1393) when treatment is adjusted demonstrating no significant association when adjusted for treatment with IVIG or rituximab. Among patients with low grade B-cell lymphoma, the incident rates of infections for patients with baseline IgG<500 mg/dL was 0.511 (95% CI 0.209, 1.248) times lower than those with baseline IgG>500 mg/dL when adjusted for treatment with IVIG or rituximab (p=0.1406). Additionally, there was no statistically significant correlation with COVID infections when evaluating grade of lymphoma (p=0.8527) or IgG level (p=0.6535). There was also no statistically significant correlation (p=0.3291) of COVID infections when treating with IVIG, rituximab, or both (**Table 4**).

### Mortality and overall survival

Patients with high grade B-cell lymphoma had a 4.584 (95% CI 2.051, 11.13) times higher death rate when compared to patients with low grade B-cell lymphoma regardless of baseline IgG level (p=0.0003) (**Table 3**). Patients with a baseline IgG level below 500 mg/dL had a 3.325 (95% CI 1.258, 9.491) times higher death rate when compared to patients with a baseline IgG level >500 mg/dL regardless of grade of B-cell lymphoma (p=0.012663) (**Table 3**). When analyzing three-year overall survival, patients with high grade B-cell lymphoma had a 0.168 (95% CI 0.06, 0.424) times lower likelihood of being alive when compared to patients with low grade B-cell lymphoma regardless of baseline IgG level (p=0.0003) (**Table 3**). Patients with a baseline IgG<500 mg/dL had a three-year overall survival that was not statistically significant (p=0.684391) regardless of grade of B-cell lymphoma (**Table 3**). When analyzing five-year overall survival, patients with high grade B-cell lymphoma had a 0.087 (95% CI 0.022, 0.297) times lower likelihood of being alive when compared to low grade B-cell lymphoma regardless of baseline IgG level (p=0.0002) (**Table 3**). Patients with a baseline IgG<500 mg/dL had a five-year overall survival that was not statistically significant regardless of grade of B-cell lymphoma (p=0.4591). For individuals who had a baseline IgG level below 500 mg/dL, mortality was evaluated. Patients with a baseline IgG level below 500 mg/dL with high grade B-cell lymphoma had a 4.8 (95% CI 1.904, 13.81) times higher death rate when compared to patients with a baseline IgG level < 500 mg/dL with low grade B-cell lymphoma (p=0.012758). When analyzing three-year overall survival, patients with an IgG level below 500 mg/dL with high grade B-cell lymphoma had a 0.213 (95% CI 0.069, 0.589) times lower three-year survival when compared to individuals with a baseline IgG level below 500 with low grade lymphoma (p=0.942711). When analyzing five-year overall survival, patients with a baseline IgG level below 500 mg/dL with high grade B-cell lymphoma had a 0.134 (95% CI 0.028, 0.537) times lower five year survival when compared to individuals with a baseline IgG level below 500 mg/dL with low grade B-cell lymphoma (p=0.545258). Multivariate logistic models accounting for IVIG or Rituximab were unable to be completed due to small sample size for three-year overall survival, five-year overall survival, and mortality.

## Discussion

To our knowledge, this is the first study to compare incidence and associated outcomes of hypogammaglobulinemia at diagnosis across aggressive and indolent NHL. Within newly diagnosed patients, 13.5% had hypogammaglobulinemia prior to intervention. Our rate of hypogammaglobulinemia was similar to the 13.5% previously reported in DLBCL (8). Results showed a 3.981 times higher rate of hospitalization (p=0.0007) and a 3.018 times higher rate of mortality (p=0.015) in B-cell NHL patients with baseline IgG <500 mg/dL (**Table 5**). Although a higher rate of hospitalization might be expected in high versus low grade lymphoma, this effect was further intensified when stratifying high grade B-cell lymphoma plus baseline IgG <500 mg/dL at 9.625 (2.371, 65.138) times higher than that in patients with baseline IgG >500 (p=0.0252). We also found 9.924 (3.152, 31.249) times higher rate of infection in high grade B-cell lymphoma with hypogammaglobulinemia compared to low grade B-cell lymphomas. These findings are consistent with prior literature in DLBCL which found that those patients with hypogammaglobulinemia had higher stage, IPI, and LDH compared to patients without hypogammaglobulinemia (9). Unfortunately due to the retrospective nature of the present study, variables for prognostication were missing in many patients and were not further analyzed. Although there was no difference in three- or five-year OS, we expect this may be due to the relatively short follow-up interval in the present study as only 114 and 54 patients were analyzed 3 and 5 years after diagnosis, respectively.

**Table 5:** Rates of infection and mortality in IgG low (<500 mg/dL) versus IgG high (>500 mg/dL).

Outcome	Estimated Odds Ratio & 95% CI
COVID Infections	0.774 (0.218, 2.158)
**Hospitalized**	**3.981 (1.812, 9.037)**
**Dead**	**3.018 (1.195, 7.22)**
Alive at 3 years	0.903 (0.329, 2.755)
Alive at 5 years	2.292 (0.704, 7.99)

The bold values indicate significant findings.

Additionally, the literature shows that absolute lymphocyte count at diagnosis portends better overall survival in follicular lymphoma, independent of the follicular lymphoma international prognostic index (FLIPI) (2). Another study found absolute lymphocyte count at diagnosis predicted event free survival and overall survival in DLBCL, regardless of front-line chemotherapy regimen (1). The association between absolute lymphocyte-monocyte ratio on PFS and OS in DLBCL is also independent of IPI (3). The authors also showed that absolute lymphocyte count predicts response to rituximab, cyclophosphamide, doxorubicin hydrochloride, vincristine sulfate, prednisone (R-CHOP).

Anti-CD20 monoclonal antibodies, such as rituximab, ofatumumab, and obinutuzumab improve outcomes in B-cell malignancies (10–16). Antibody therapies are associated with hypogammaglobulinemia and recurrent infections in 14-50% of patients (17–21). Rituximab is well known to cause hypogammaglobulinemia in as many as 39% of B-cell lymphoma patients with normal IgG levels prior to therapy (17). One study of rituximab in 310 patients with follicular lymphoma found antibody-induced hypogammaglobulinemia in 74.4% in chemotherapy-naïve patients who either received anti-CD20-based therapy or no systemic therapy (22). Of patients who received anti-CD20 antibody, 45.2% developed low IgG. Hypogammaglobulinema has been associated with cumulative dose of anti-CD20 antibodies (17, 23); the proposed mechanism through permanent damage of B-cell maturation and blocking of plasma cell differentiation (18). Previous studies have linked prolonged antibody-induced hypogammaglobulinemia with low baseline serum immunoglobulin level with OR 4.2 (95% CI 1.26-14.1) (24, 25). Another review found antibody-induced hypogammaglobulinemia in 39% of patients on active treatment with rituximab and 54% of patients on maintenance therapy (17). In Casulo 2013, 15% of patients had pre-rituximab hypogammaglobulinemia, of which 72% had further decreasing levels of immunoglobulins post treatment (17). The findings in the present study are consistent with existing literature which showed rituximab only increased risk of grade ≥3 infections in advanced-stage Hodgkin lymphoma (26). The authors hypothesize that although CD20 is expressed in increasing concentration during normal and malignant B-lymphocyte maturation, it is absent on pro-B cells and plasma cells explaining why rituximab does not directly impair immunoglobulin production (27). Per current guidelines, patients with recurrent bacterial infection or severe bacterial infections with IgG <500 mg/dL may be supplemented with IVIG. Recurrent sinopulmonary infections requiring IVIG occurred in 6.6% of participants (17).

Although other studies have shown hypogammaglobulinemia across multiple subtypes of NHL (5, 8, 22), this study focused on IgG isotype due to its clinical and therapeutic significance. T cell lymphomas were excluded from the present study given prior studies have not found an association with hypogammaglobulinemia (5). Hypergammaglobulinemia has also been reported in approximately 17% of NHL patients though the clinical significance of this is unclear (28). The authors did not find a significant association between immunoglobulin concentration and either malignancy grade or immunologic origin of lymphoma.

Baseline levels were recorded around the time of NHL diagnosis, thus we cannot conclude if immunoglobulin levels were decreased prior to development of NHL. Given that decreased immunoglobulin levels were seen across disease subtypes, we hypothesize decreased immunoglobulin levels were the result of underlying lymphoma. Lymphoproliferative disorders are well established as a consequence of one of the most common forms of immunodeficiency, Common Variable Immunodeficiency (CVID), including lymphoma in up to 8% of patients (29). CVID patients are also at increased risk of lymphoproliferative disorders other than lymphoma such as lymphadenopathy and lymphoid hyperplasia (29). Within our study population, only 1 of our 223 patients was diagnosed with CVID. The etiology between primary hypogammaglobulinemia (CVID-associated lymphoma) and secondary (lymphoma-associated) hypogammaglobulinemia is thought to be due to immune dysregulation and defective B-cells which cannot produce plasma cells which make immunoglobulins. The association between an altered immune system and lymphoma has been well established. In CLL, the hypogammaglobulinemia is thought to be due to dysfunction of the non-clonal CD5-negative B-cells (30) and is more severe with longer disease duration and more advanced disease (31). One survey of the cBioPortal of 50 genes commonly altered in CVID found these genes prevalent in 25% of 1,309 NHL samples analyzed (32). Recent studies have brought into question if lymphoma patients with hypogammaglobulinemia should be reclassified as having CVID given similar clinical and molecular characteristics (33).

Limitations of this study include retrospective nature, short-follow-up period, and variation in IgG measurement practices at diagnosis.

## Conclusions

This study analyzed incidence of hypogammaglobulinemia across aggressive and indolent B-cell NHL and associated clinical outcomes. These findings demonstrate an association between hypogammaglobulinemia and rate of infection and rate of hospitalization across the spectrum of B-cell NHL. This effect was further amplified in patients with aggressive B-cell lymphoma. Given the common use of monoclonal antibodies to treat B cell lymphomas of all subtypes and the known risk of hypogammaglobulinemia from these treatments and the common finding of hypogammaglobulinemia at diagnosis of B cell NHL, pretreatment screening of IgG levels may be important to identify patients at risk for infection, hospitalization and death. Studies of intervention with IgG supplementation in such patients are needs to learn if such complications can be mitigated with treatment.

## Data availability statement

The raw data supporting the conclusions of this article will be made available by the authors, without undue reservation.

## Ethics statement

The studies involving humans were approved by University of California Irvine IRB. The studies were conducted in accordance with the local legislation and institutional requirements. Written informed consent for participation was not required from the participants or the participants’ legal guardians/next of kin in accordance with the national legislation and institutional requirements.

## Author contributions

DB: Data curation, Formal analysis, Investigation, Methodology, Writing – original draft, Writing – review & editing. CG: Data curation, Formal analysis, Writing – original draft, Writing – review & editing. AC: Data curation, Writing – review & editing. W-PC: Formal analysis, Writing – review & editing. LP-B: Conceptualization, Methodology, Supervision, Writing – review & editing.
